# Annotations

**Published:** 1907-09-14

**Authors:** 


### Annotations.

### The Binaural Stethoscope.

It is with much satisfaction that we have read Sir Douglas Powell's endorsement of the acoustic limitations of the binaural stethoscope. This form of stethoscope is so convenient, and has other advantages of its own, that it has very largely replaced the rigid instruments, and many practitioners, we believe, never employ the latter in any circumstances. Sir Douglas points out that the flexible stethoscope is a very inadequate medium for the appreciation of aortic shock sounds and impulses, which, even when quite impalpable, can often be appreciated through the rigid instrument. With the flexible stethoscope, again, conducted oral breath sounds are not unlikely to be mistaken for morbid sounds of pulmonary origin. Personally, we think it necessary to go further than this, and to say that there are certain cardiac murmurs, and even some abnormal pulmonary sounds, which are apt to be missed in binaural examination though they are readily detected both on direct auscultation or by the use of a rigid stethoscope. Hence it seems necessary to recognise the rigid stethoscope as the one with which in questions of auscultation the final verdict lies. The difficulty in using sometimes one and sometimes the other is the maintenance by the physician, in his own mind, of two standards, but even this is not impossible. And as each type of instrument has its own value, such a demand seems to be necessary. The important thing, however, just now is the formal proclamation that the flexible stethoscope is not a completely satisfactory substitute for the original form of instrument. It has its value, but it has also its limitations. A point worth noting in some of the contributions on this subject is the occasional confusion of the binaural with the "double" stethoscope. The binaural, of course, is not a "double" stethoscope, but a single stethoscope with two ear-pieces. The true "double" stethoscope is that of Dr. Scott Alison, with its two flexible stems connected by a joint, and each having its own ear-piece and its own receiver or chest piece.

### The Food of French Children.

At a time like the present, when so much attention is being given to the feeding of young children, it is a curious example of the different ideas of different nations to read the account of the bringing up of French children given by Miss Betham Edwards in her book on "Home Life in France." The French child is an important personage, for the parents, although, or because, they limit the size of the family so determinedly, make much of the children they have. Therefore their ideas of feeding, however remarkable they may seem to us, do not spring from carelessness. Certainly the French infant gets a better start in life than many English ones, because he is almost sure to have a wet-nurse, a healthy peasant, so that the risks which are run by the "bottle baby" are practically unknown. But after this stage the French child, however young, shares the ordinary food of the family, which is generally more elaborate than that of the corresponding class in England. Miss Betham Edwards gives the menu provided for a two and half year old girl whom she knew: "Soup, fish, beef steak, fried potatoes, cheese, dessert, and, of course, wine." All French children drink wine, and apparently the idea of it being bad for them either physically or morally never enters their parents' heads. For the rest, "everything that French babies like is supposed to be good for them," says Miss Betham Edwards, "and as the national physique is noted for its elasticity and powers of resistance, there may be practical wisdom in thus eschewing a nursery diet." So says this amiable English critic, but against her opinion we may set the fact that of all things English the French admire nothing so much as our children, their rosy faces and their sturdy limbs, while to the average English eye French children seem sallow and unwholesome. They may survive the "confused feeding" of their childhood, and as whatever they get is likely to be well-cooked their chances are better than would be those of an English child brought up on an equally varied diet, but we can hardly be convinced that they thrive upon it, and that they would not be better with simpler fare.

### The Effect of One Disease upon Another.

In the *Medical Chronicle* for August Dr. Judson Bury describes two cases of interest in themselves, and not devoid of significance in relation to the practice of therapeutics. The first patient was a man of 44 years, extremely ill with the general evidences of severe cardiac failure, probably due to chronic alcoholism. The symptoms extended over several weeks in spite of treatment, and caused necessarily a most anxious outlook. Following a hypodermic injection of strychnine into the arm, cellulitis developed and involved the whole of the limb. Yet two days after the first sign of this condition the patient's general condition began to improve—the breathing became easier, the pulse slower and stronger; and gradually all the alarming symptoms subsided, and the man left the hospital in apparent good health. Dr. Judson Bury's suggestion is that the cardiac failure following chronic alcoholism may be due, not to the direct action of alcohol on the cardiac muscle, but to the influence of some unknown toxin which the weakened muscle is unable to resist. Hence in such a case as the above it may be supposed that the attack of cellulitis led to the development of appropriate antitoxins which neutralised the toxins engaged in prejudicing cardiac activity. In the second case the patient was the subject of an obscure febrile illness, followed by widespread evidences which were interpreted as those of disseminated sclerosis. In this condition he suffered from small-pox, and with the onset of this disease his nervous symptoms gradually disappeared excepting only atrophy of one half of the tongue. Here again some such suggestion as that adopted in the first case may be applied. Of course it may be said that exceptional cases similar to the above occur without the intervention of a second disease. That is true. But the question remains, Why? Is it possible that by some other means there may be produced in the body the efficient antitoxins which in Dr. Judson Bury's cases were called into being by the agency of a new morbid influence?

